# Tunnel injection from WS_2_ quantum dots to InGaN/GaN quantum wells

**DOI:** 10.1039/c7ra13108a

**Published:** 2018-04-24

**Authors:** Svette Reina Merden Santiago, Septem P. Caigas, Tzu-Neng Lin, Chi-Tsu Yuan, Ji-Lin Shen, Ching-Hsueh Chiu, Hao-Chung Kuo

**Affiliations:** Department of Physics and Center for Nanotechnology, Chung Yuan Christian University Chung-Li 32023 Taiwan jlshen@cycu.edu.tw; Department of Electronic Engineering, Chung Yuan Christian University Chung-Li 32023 Taiwan; Department of Photonics and Institute of Electro-Optical Engineering, National Chiao-Tung University Hsinchu 300 Taiwan

## Abstract

We propose a tunnel-injection structure, in which WS_2_ quantum dots (QDs) act as the injector and InGaN/GaN quantum wells (QWs) act as the light emitters. Such a structure with different barrier thicknesses has been characterized using steady-state and time-resolved photoluminescence (PL). A simultaneous enhancement of the PL intensity and PL decay time for the InGaN QW were observed after transfer of charge carriers from the WS_2_-QD injector to the InGaN-QW emitter. The tunneling time has been extracted from the time-resolved PL, which increases as the barrier thickness is increased. The dependence of the tunneling time on the barrier thickness is in good agreement with the prediction of the semiclassical Wentzel–Kramers–Brillouin model, confirming the mechanism of the tunnel injection between WS_2_ QDs and InGaN QWs.

## Abstract 2

In this manuscript, an effective tunnel-injection structure, in which the WS_2_ quantum dots (QDs) act as the electron injector and the InGaN quantum wells (QWs) act as the light emitters, separated by GaN barriers.

## Graphical abstract

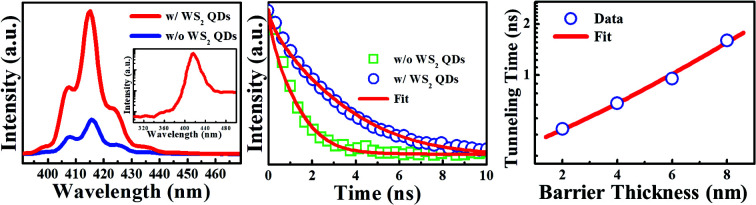

## 1. Introduction

Development of semiconductor heterostructures for applications in devices such as light-emitting diodes (LEDs), phototransistors, and photovoltaics is of great interest in research today.^1,2^ One of the recent interests in semiconductor heterostructures is the incorporation of two-dimensional materials with conventional semiconductors.^3,4^ Carrier or energy transfer between two-dimensional materials and semiconductors was proven to be promising for the enhancement of electrical and optical properties of the semiconductor heterostructures. Among them, the graphene-semiconductor heterostructure is regarded as one of the most promising structures for developing high performance electronics and optoelectronics.^5–7^ For example, a photodetector that integrated graphene and PbS quantum dots (QDs) has been demonstrated to achieve high charge collection efficiency and ultra-high gain.^5^ Also, a GaN nanorod-graphene hybrid device structure exhibited enhancements of photocurrent, sensitivity, and photoresponsivity.^6^

In addition to graphene, other two-dimensional materials such as transition metal dichalcogenide (TMD) compounds have been discovered subsequently and have attracted a lot of attention. Unlike graphene, TMD compounds with strong in-plane bonding and weak out-of-plane interactions are non-centrosymmetric. Because of novel physical and electronic properties, TMD compounds have been used in a wide range of applications such as electronics, optoelectronics, sensing, lubrication, catalysis, energy storage, and bioimaging.^8–13^ Tungsten disulfide (WS_2_) is one of TMD materials, which is an anisotropic material with a trigonal prismatic structure. The band gap of WS_2_ can be tuned from indirect gap at multilayer thickness to direct gap at monolayer thickness.^14^ By minimizing the lateral size of WS_2_ nanosheets to less than 10 nm, WS_2_ QDs can be produced, which exhibit peculiar optical and electrical properties owing to high surface area and many active edge sites.

Recently, WS_2_ QDs was utilized in WS_2_ QDs-ZnO heterojunction for light emitting devices.^15^ The WS_2_ QDs-ZnO heterostructured device can emit broad white light for potential applications in optoelectronics. Here, we propose that the WS_2_ QDs can be an efficient injector in the tunnel-injection structure because two-dimensional materials have less dangling bonds on their surfaces or interfaces. This is advantageous for the tunnel injection structure since a high-quality interface produces an efficient carrier transfer between the injector and the light emitter. In this study, we investigated the tunnel injection from WS_2_ QDs to InGaN/GaN quantum wells (QWs) for developing the active region in the light emitting devices. The spatial separation between the injector (WS_2_ QDs) and the light emitter (InGaN QWs) was controlled by a potential barrier layer (GaN), where the barrier layer has different thicknesses ranging from 2 to 8 nm. The carriers generated from WS_2_ QDs were demonstrated to be injected into the InGaN QW through tunneling, enhancing the light emission from the InGaN QW. The steady-state and time-resolved photoluminescence (PL) of the InGaN QW as a function of the barrier thickness were investigated and analyzed by the Wentzel–Kramers–Brillouin (WKB) approximation.

## 2. Experimental section

The WS_2_ QDs (injectors) studied were synthesized using the pulsed laser ablation (PLA) method, which has been described elsewhere. Here, a 0.03 g of WS_2_ ultrafine powder, purchased from Graphene Supermarket, was dissolved in ethanol of 7 mL *via* a vortex shaker with an angular velocity of 6000 rpm. The mixture solution of 600 μL was deposited in a quartz bottle and irradiated by an optical parametric oscillator (OPO) laser (EKSPLA NT242A) with an excitation wavelength of 415 nm and a frequency of 10 Hz. The OPO laser was controlled under the fluence of ∼40 mJ for 30 min. Concurrent with pulsed laser ablation, continuous mixing of the solution was carried out by a rotator with an angular velocity of 80 rpm. Following PLA, the suspension was subjected to centrifuge at 6000 rpm for 60 min for separation of precipitate and supernatant, where the supernatant (WS_2_ QDs) was collected subsequently. The WS_2_ QDs were assumed to be n-type since the WS_2_ nanosheets are n-type semiconductors intrinsically.^16^

The InGaN/GaN QW (light emitter) was grown by metal–organic chemical vapor deposition on a sapphire (0001) substrate. A single 2 nm-thick QW was grown after the growth of a 2 μm-thick GaN buffer layer. To adjust the spatial separation between WS_2_ QDs and InGaN QWs, the GaN barrier layer (spacer layer, *d*_sp_) were grown with four different thicknesses (2, 4, 6, and, 8 nm). To examine the tunnel injection in the WS_2_ QD-InGaN QW structure, the synthesized WS_2_ QDs was deposited on top of the QW sample *via* dropcasting.

Transmission electron microscopy (TEM) (JEOL JEM-2100F) and X-ray photoelectron spectroscopy (XPS) (Thermo Scientific K-Alpha ESCA instrument) equipped with a monochromatized Al-Kα X-ray source at 1486.6 eV were used to analyze the morphology and chemical component of WS_2_ QDs, respectively. Jasco V-750 UV-Visible spectrophotometer and Horiba Jobin Yvon FluoroMax-4PL spectrometer were used to obtain the absorbance and room-temperature PL spectra of WS_2_ QDs, respectively. A pulsed laser with a wavelength of 260 nm, duration of 250 fs, and repetition frequency of 20 MHz was used as the excitation source for the steady-state and time-resolved PL measurements of the tunnel injection from WS_2_ QDs to InGaN/GaN QWs. The collected PL was imaged through a 0.75 m grating monochromator and detected with a high-speed photomultiplier tube (PMT). The technique of time-correlated single-photon counting (TCSPC) was used for detecting the time-resolved PL signal. TCSPC creates decay transients by detecting the PL from InGaN/GaN QWs under a fixed emission wavelength, producing a histogram on a basis of photon intensity. The instrument response of TCSPC system is around 200 ps.

## 3. Results and discussion

**Fig. 1:**
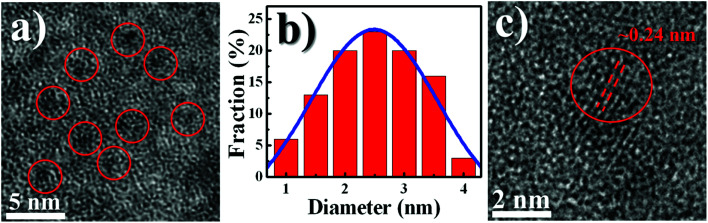
(a) TEM image, (b) Gaussian distribution of average diameter, and (c) HRTEM images of WS_2_ QDs synthesized by pulsed laser ablation (PLA).

Fig. 1a shows the TEM image of WS_2_ QDs, demonstrating that WS_2_ QDs are dispersed with a size distribution ranging from 1 to 4 nm. An average diameter of WS_2_ QDs was found to be 2.5 ± 0.4 nm according to the Gaussian fit as depicted in Fig. 1b. This average size is comparable with the result of Ghorai *et al.*, where the size of their WS_2_ QDs produced by prolonged sonication is around 3.5 nm.^15^ The high resolution TEM (HRTEM) image of WS_2_ QDs, shown in Fig. 1c, displays a lattice spacing of ∼0.24 nm, in agreement to the earlier work in Lin *et al.*^17^

**Fig. 2:**
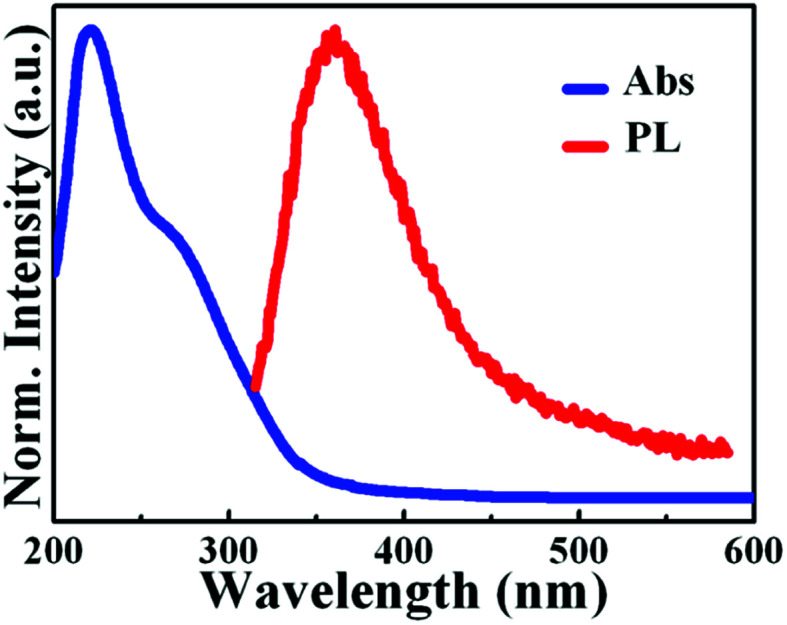
Absorbance and photoluminescence (PL) spectra of WS_2_ QDs.

Fig. 2 displays the data of the absorption and PL spectra of synthesized WS_2_ QDs. The absorption spectrum demonstrates a peak at ∼230 nm and a shoulder at ∼290 nm. The cutoff of wavelength at 200 nm is due to instrument limit of detection in the spectrophotometer. The peak at ∼230 nm is attributed to the strong quantum confinement effect due to the small size of the WS_2_ QDs.^18–20^ While the shoulder peak at ∼275 nm is attributed to optical transitions between the density of states in the valence and conduction band.^16^ Previous studies have reported that WS_2_ QDs, synthesized through different methods, emit blue luminescence mostly. Likewise, WS_2_ QDs synthesized *via* PLA exhibit an emission peak at 360 nm under the excitation wavelength of 300 nm, as shown in Fig. 2.

**Fig. 3:**
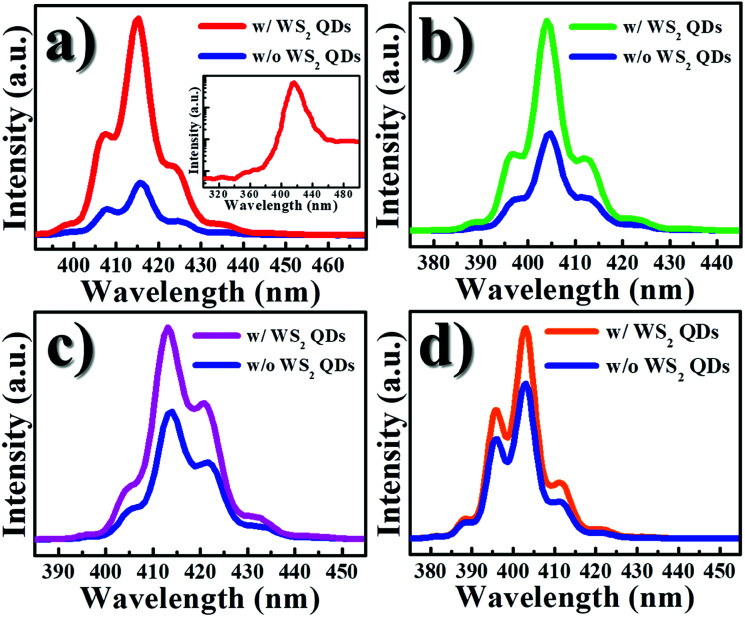
PL spectra of bare InGaN/GaN QWs (blue line) and InGaN/GaN QWs with WS_2_ QDs. The cap layer thickness (barrier) of InGaN/GaN QW is (a) 2 nm; (b) 4 nm; (c) 6 nm; and (d) 8 nm, respectively. The inset in (a) shows the semilog plot of the PL spectrum in InGaN/GaN QWs with WS_2_ QDs ranging from 300 to 500 nm.

**Fig. 4:**
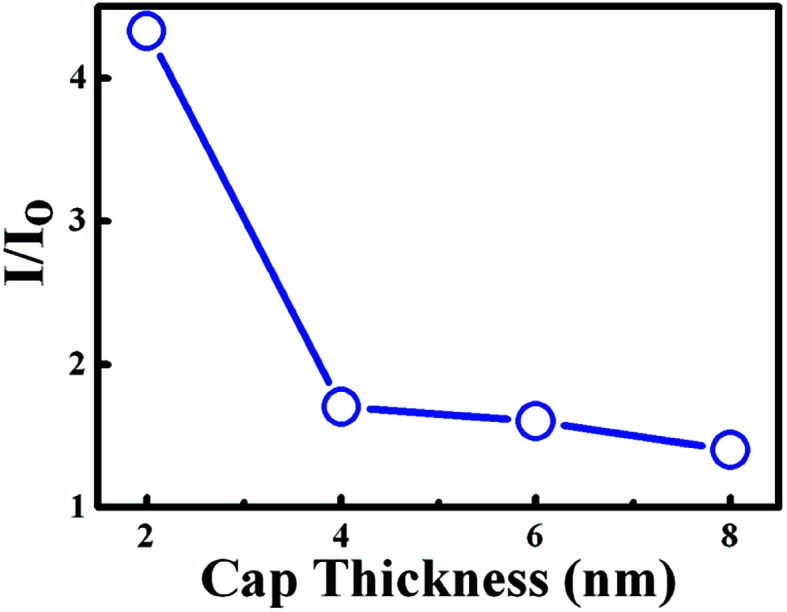
PL intensity ratio of InGaN/GaN QWs with WS_2_ QDs to that of bare InGaN/GaN QWs as a function of cap thickness. The line is a guide for the eye.

Fig. 3 displays the PL spectra of the bare InGaN QW and the InGaN QW with WS_2_ QDs, where the thickness of *d*_sp_ ranges from 2 to 8 nm. After introduction of WS_2_ QDs, an increase of the PL intensity in the InGaN QW was observed. Fig. 4 shows the PL intensity ratio of the InGaN QW with WS_2_ QDs to that of without WS_2_ QDs as a function of *d*_sp_. The enhancement of PL intensity reveals a 4.3, 1.7, 1.6, and 1.4 fold for the InGaN-QW sample with *d*_sp_ = 2, 4, 6, and 8 nm, respectively. The cap thickness (*d*_sp_) thus plays an important role for controlling the charge carriers in the InGaN QW since the PL intensity is associated with the charge carrier density. It is noted that the PL from WS_2_ QDs is unnoticeable for the InGaN QW with WS_2_ QDs, as shown in the inset of the Fig. 3(a). The PL in WS_2_ QDs is supposed to appear at 360 nm (from Fig. 2), but no obvious PL signal was observed at that wavelength. The PL emitted from WS_2_ QDs could be merged by the strong PL of the InGaN QW, which has high luminescence efficiency.

**Fig. 5:**
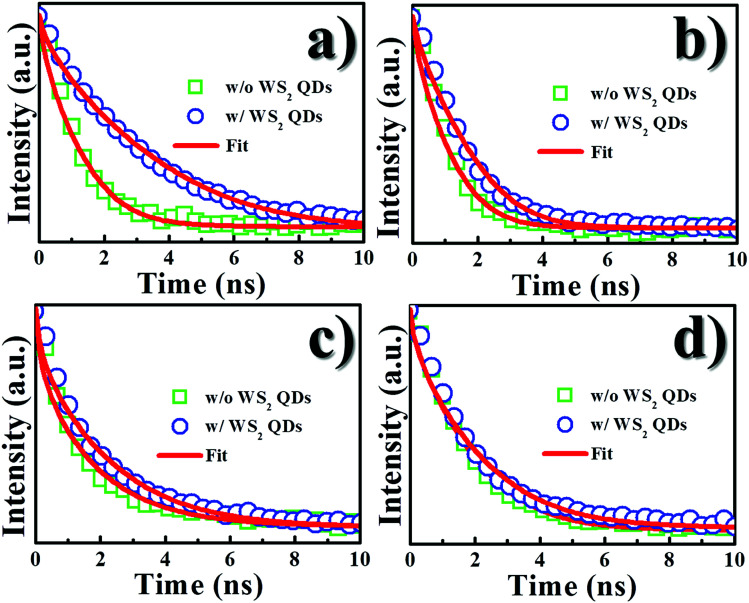
PL transients of bare InGaN/GaN QWs (green squares) and InGaN/GaN QWs with WS_2_ QDs (blue circles) with the cap thickness of (a) 2 nm; (b) 4 nm; (c) 6 nm; and (d) 8 nm. Red lines represent fits of simulated data using eqn (1).

To further explore the carrier dynamics in InGaN QWs, time-resolved PL measurements were performed. Fig. 5(a)–(d) represents the PL transient of the InGaN QW with WS_2_ QDs having a spacer thickness of 2, 4, 6, and 8 nm, respectively, under a detection wavelength at the PL peak. The open squares represent bare InGaN QW while the open circles represent InGaN QW with the injection of WS_2_ QDs. It was found the PL transients of the InGaN QWs with WS_2_ QDs decay less as compared with that in bare InGaN QWs. The red lines in Fig. 5 represent the fits of PL decay curves using a stretch exponential function:^21^

1 *n*(*t*) = *n*(0)exp[−(*kt*)^*β*^]

where *n*, *k* and *β* represent the carrier densities, the decay rate and the dispersive component, respectively. In a stretched exponential function, the average decay time *τ*_PL_ can be obtained by:^22^2
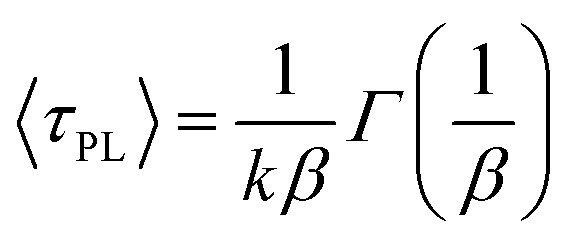
where *Γ* is the Gamma function. The calculated *β*, *k*, and 〈*τ*_PL_〉 from the PL decays in Fig. 5 are shown in Table 1. It was found the difference of average decay time between the InGaN QW associated with WS_2_ QDs and the bare InGaN QW is pronounced with decreasing *d*_sp_. This gives an evidence that the increased carriers are injected into the InGaN QWs from WS_2_ QDs, leading to an increase of the PL decay time. The amount of injected carriers enhances more pronouncedly as the barrier thickness decreases. Thus, the decay of PL reflects the competition between two processes: the recombination in the QW and the carrier transfer from WS_2_ QDs.

**Table 1:** The calculated dispersion component (*β*), decay rates (*k*), and average decay time (*τ*_PL_)

Barrier thickness (nm)	Without WS_2_ QDs			With WS_2_ QDs		
	*β*	*K* (ns^−1^)	*τ*_PL_ (ns)	*β*	*K* (ns^−1^)	*τ*_PL_ (ns)
2	0.65	4.545	0.301	0.67	1.667	0.793
4	0.67	4.76	0.278	0.64	2.123	0.471
6	0.48	7.69	0.281	0.535	4.347	0.401
8	0.68	5.26	0.247	0.59	5.26	0.292

**Fig. 6:**
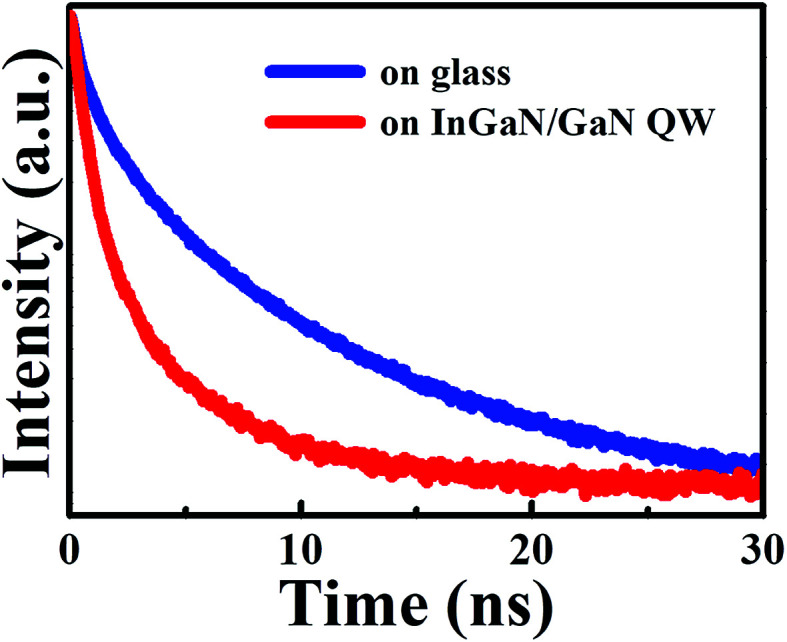
PL transients of WS_2_ QDs on a glass substrate and on the InGaN/GaN QW sample (cap thickness = 2 nm).

To find out whether the increased carriers in the InGaN QW stemmed from WS_2_ QDs, the PL dynamics of WS_2_ QDs was investigated. Fig. 6 shows the PL decay transients of WS_2_ QDs deposited on a glass and on the InGaN/GaN QW (barrier thickness = 2 nm) under the detection at the emission wavelength of 420 nm. The PL decay time of WS_2_ QDs on the InGaN/GaN QW was found to decrease drastically compared to that on the glass. This indicates the carriers in WS_2_ QDs on the InGaN/GaN QW have escaped out of QDs efficiently, producing a reduction of PL decay time. The decrease of the PL decay time in WS_2_ QDs concurrent to the increase of PL decay time in InGaN/GaN QW reveals that carrier transfer may take place from WS_2_ QDs to InGaN/GaN QW.

**Fig. 7:**
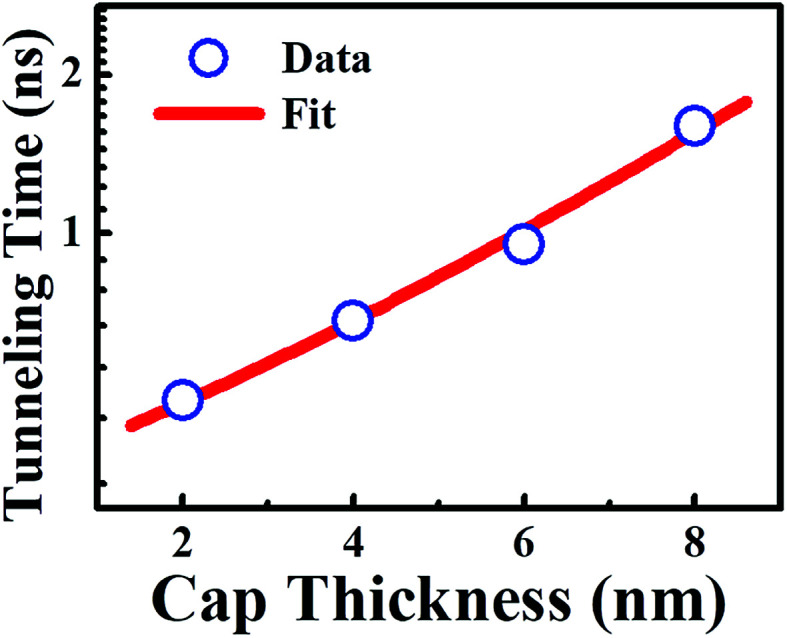
Tunneling time calculated from the PL transient as a function of the cap thickness. Blue circles represent calculated data and red line represents the result of fits with WKB approximation (eqn (4)).

From the above data (Fig. 3, 5 and 6) we suggest that the enhanced PL intensity and PL decay time in the InGaN QW are attributed to the transfer of the photogenerated carriers from WS_2_ QDs *via* tunnel injection. Thus, the carrier tunneling time *τ*_tun_ can be estimated by considering the total decay rate, *τ*_PL_^−1^, is the sum of recombination decay rate, *τ*_rec_^−1^, and the rate of carrier tunneling, *τ*_tun_^−1^, using the following equation:3
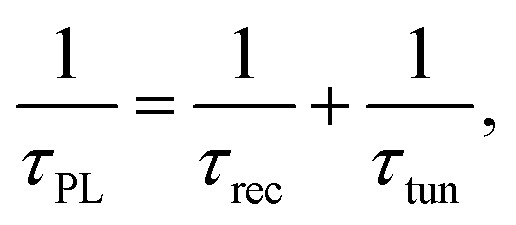
where *τ*_PL_ is the measured PL decay time and *τ*_rec_ is the recombination time. From *τ*_PL_ of InGaN QWs without and with WS_2_ QDs as well as eqn (3), *τ*_tun_ was determined and displayed in Table 2. Fig. 7 displays the tunneling time as a function of barrier width, revealing an increase in the barrier thickness leads to an increase in the tunneling time.

**Table 2:** The calculated decay rates of InGaN/GaN QW, hybrids and tunneling time *vs.* cap (barrier) thickness

Barrier thickness	*τ*_PL_	*τ*_rec_	*τ*_tun_
2	0.301	0.793	0.48
4	0.278	0.471	0.69
6	0.281	0.409	0.95
8	0.247	0.292	1.6

The dependence of the tunneling time on the barrier thickness may be comprehended with a semiclassical WKB model. In the WKB approximation, the tunneling time increases exponentially with the barrier thickness using the following relation:^23,24^4
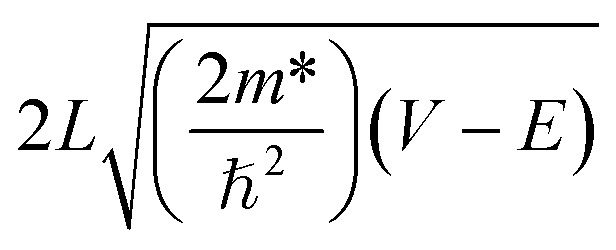
where *L* is the barrier thickness, m* is the effective mass in the barrier layer, *V* is the band discontinuity of the conduction band (well depth), and *E* is the lowest confinement energy level in the QW. This equation predicts a nearly linear relationship between *τ*_T_ and *L*. The dependence of the tunneling time *τ*_tun_ on the barrier thickness was analyzed using the WKB approximation and shown as the solid line in Fig. 7. A good fit to the experimental dependence of *τ*_T_ on the *L* is found. This confirms that mechanism of the carrier transfer from WS_2_ QDs into InGaN QWs is governed by the tunneling. An increase in the barrier thickness leads to an increase of tunneling time, implying the tunneling coupling decreases as the barrier width increases. Similar results have been reported previously with regard to the carrier transfer *via* tunneling.^24–26^

To find out processes of the tunnel injection from WS_2_ QDs to InGaN/GaN QWs, the band diagram of InGaN/GaN QW was analyzed using APSYS simulation program,^27^ while the work functions of WS_2_ QDs as well as the GaN layer were measured using Kelvin probe. For the work function measurement, the contact potential difference (VCPD) between the sample and tip which is related to the difference in work function between the two is described by:^26^

**Fig. 8:**
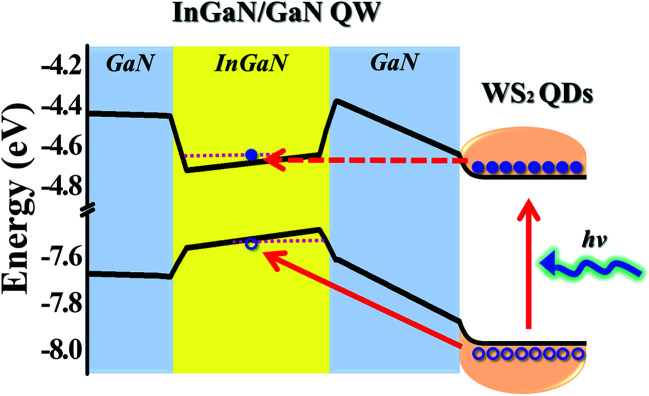
Proposed diagram of carrier transfer *via* tunneling from WS_2_ QDs to InGaN/GaN QWs.

5 e*V*_CPD_ = *W*_tip_ − *W*_sample_,

where *W*_tip_ and *W*_sample_ represents work functions of the tip and sample, respectively. The *W*_sample_ can then be determined by calibration of *W*_tip_ and *V*_CPD_ measurements. Using eqn (5), the work function of WS_2_ QDs was determined to be 4.77 ± 0.02 eV. While the work function of the GaN that serves as the barrier between WS_2_ QDs and InGaN QWs is 4.38 ± 0.03 eV. Based on the work function results, we propose an energy diagram for the tunnel injection mechanism from WS_2_ QDs to the InGaN QW, as displayed in Fig. 8. Under photoexcitation with the photon energy greater than that of the bandgap in WS_2_ QDs, electrons from QDs are generated. The WS_2_ QDs can act as energy injectors, transferring their photogenerated electrons into the InGaN QW *via* a tunneling process (the dashed arrow). Concurrently, the photogenerated holes in WS_2_ QDs can move into the InGaN QW since the valence band in InGaN QW has a lower energy state compared to that in WS_2_ QDs. When the transferred electrons and holes recombine in the InGaN QW, the PL intensity and PL decay time of the InGaN QW (the light emitter) are enhanced accordingly. The efficient tunneling injection from WS_2_ QDs to InGaN QWs demonstrates that the tunnel-injection nanostructures including WS_2_ QDs and InGaN QWs could be a candidate for realizing high-efficiency light emitting devices.

## 4. Conclusion

We have investigated a tunnel-injection structure including the injector of WS_2_ QDs, the barrier of GaN layers, and the light emitter of InGaN QWs. Steady state and time-resolved PL were used to characterize the tunnel injection process. A simultaneous increase in the PL intensity and PL decay time in InGaN QWs were observed in the presence of WS_2_ QDs. The tunneling time as a function of the barrier thickness has been determined, which agrees well with the WKB approximation. This study reveals that the tunnel-injection structure containing WS_2_ QDs and InGaN QWs is promising for their potential applications in the design of active layer in light emitting devices.

## Conflicts of interest

There are no conflicts of interest to declare.

## Supplementary Material
