# Correction: Tunnel injection from WS_2_ quantum dots to InGaN/GaN quantum wells

**DOI:** 10.1039/c8ra90034e

**Published:** 2018-05-03

**Authors:** Svette Reina Merden Santiago, Septem P. Caigas, Tzu-Neng Lin, Chi-Tsu Yuan, Ji-Lin Shen, Ching-Hsueh Chiu, Hao-Chung Kuo

**Affiliations:** Department of Physics, Center for Nanotechnology, Chung Yuan Christian University Chung-Li 32023 Taiwan jlshen@cycu.edu.tw; Department of Electronic Engineering, Chung Yuan Christian University Chung-Li 32023 Taiwan; Department of Photonics, Institute of Electro-Optical Engineering, National Chiao-Tung University Hsinchu 300 Taiwan

## Abstract

Correction for ‘Tunnel injection from WS_2_ quantum dots to InGaN/GaN quantum wells’ by Svette Reina Merden Santiago *et al.*, *RSC Adv.*, 2018, **8**, 15399–15404.

Eqn (4) in the published paper was incorrect; the correct version is shown below:4
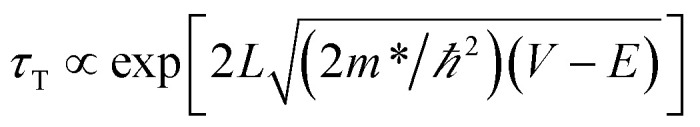


The Royal Society of Chemistry apologises for these errors and any consequent inconvenience to authors and readers.

## Supplementary Material

